# Challenges and strategies of children and adolescents with inflammatory bowel disease: a qualitative examination

**DOI:** 10.1186/1477-7525-5-28

**Published:** 2007-05-25

**Authors:** David B Nicholas, Anthony Otley, Claire Smith, Julie Avolio, Marla Munk, Anne M Griffiths

**Affiliations:** 1 The Hospital for Sick Children, 555 University Avenue, Toronto, Ontario, M5G 1X8, Canada; 2 IWK Health Centre, PO Box 9700 Rpo CSC, Halifax, Nova Scotia, Canada

## Abstract

**Background:**

The aims of this study were to understand the lived experience and elements of quality of life as depicted by children and adolescents with inflammatory bowel disease (IBD).

**Methods:**

Eighty participants with IBD, ranging in age from 7 to 19 years, were interviewed about the impact of IBD on their daily lives.

**Results:**

Findings demonstrated that IBD profoundly affects children and adolescents. These young patients experience concerns and discomfort as a result of IBD symptoms and treatments. They commonly feel, in varying degrees, a sense of vulnerability and diminished control over their lives and future, and perceive themselves as "different" from healthy peers and siblings. Despite these negative impacts, participants also described effective means of coping with IBD, and reported that support from family members and friends contributes to coping. A positive attitude and other strategies were also described as strengths contributing to quality of life.

**Conclusion:**

Clinical assessments need to consider the experiences and perceptions of children as they manage their IBD. Implications for clinical practice are discussed.

## Background

Inflammatory Bowel Disease (IBD) comprises two major chronic intestinal disorders of unknown etiology: Crohn's disease and ulcerative colitis. Both result in ongoing or recurring symptoms including diarrhea, pain, poor appetite and growth retardation (weight and height). IBD is reported to manifest during childhood or adolescence in 20–25% of patients [1,2]. The incidence of IBD varies globally with a lower incidence in Asian, Mediterranean and Middle Eastern countries, and a higher incidence ranging from 0.3% to 0.8% of the population in northern Europe, Scandinavia, New Zealand and the United States [3]. Pharmacologic, nutritional and surgical treatments available to patients require adherence to prescribed regimens which often necessitate adjustment in daily activity. The chronic, unpredictable gastrointestinal symptoms and complications associated with IBD, and the required treatments all impose psychological and social stresses on young patients [2].

Children with IBD may be at risk for negative psychosocial outcomes including stress [4-7], social strain [8,9], altered self image [5,6,8] and psychiatric sequelae [5,6,9]. Significant school absenteeism was reported in 60% of children within a sample of Crohn's disease patients [10], and of those children who had taken examinations, 80% believed they had underachieved as a result of their condition [10]. Maunder and Esplen [8] suggest that bowel dysfunction may result in social isolation and blame, and in some cases of surgical interventions such as ileostomy, stigma negatively affects body image. Clearly, IBD imposes frequent unpredictability which perhaps corresponds to findings suggesting that children and adolescents with IBD tend to perceive circumstances in their lives to be outside of their control [5].

Families of children with IBD also appear to be profoundly affected by the condition[11]. Engstrom [5] suggests higher levels of family dysfunction within these families relative to comparison groups of families with diabetes and healthy children. Mothers of children with IBD indicate little internal family support, and findings suggest a maternal wish that their family be happier than it is perceived to be [5].

In spite of mounting evidence suggesting a negative impact of IBD on the quality of life of young patients, little research has focused on the lived experience of children and adolescents with IBD nor on how IBD is perceived within the context of daily life. A considerable body of literature illuminates quality of life of children with chronic illness more generally [12,13], however this literature often assumes, or perhaps by default implies, homogeneity of experience among children with diverse chronic illnesses including but not exclusively IBD. Despite the potential for similarities based on factors such as (i) illness chronicity and (ii) ongoing compromised health status, specific conditions and their differing impact on family members merit research attention [14,15]. To address this gap, objectives of this paper, as a part of a larger project aimed at developing an evaluative disease-specific measure of quality of life in pediatric IBD [16] were to understand: (i) how children understand and make meaning of IBD, and (ii) how IBD affects daily life.

## Methods

In-depth interviews were conducted in person with children and adolescents with IBD. Based on an interpretive ethnographic approach [17] in-depth interviews permitted exploration of lived experience from the vantage point of children and adolescents' life world, perspectives and meanings. Participants were purposively selected for variation in age and condition from a complete database of children being treated at a central Canadian children's hospital that offers a large regional referral and treatment center for children and adolescents with Crohn's disease or ulcerative colitis. The participants interviewed were also part of a larger study [2] aimed at developing a health-related quality of life measure for children and adolescents with IBD.

Participants ranged in age from 7 to 19 years with a mean age of 13.3 years and a mode age of 16 years. 44 participants were male and 36 were female. The majority of children had Crohn's disease (N = 61) while N = 19 had ulcerative colitis, based on accepted clinical, radiologic, endoscopic and histologic criteria. The sample was ethnically diverse, and participants lived within a variety of family constellations including single parent, blended and nuclear families. In order to ensure sufficient time to adjust to their condition, all participants had been diagnosed a minimum of 6 months. Interviews were audio-recorded and took place in the participants' home or the clinic. A semi-structured interview guide, probing for participants' experiences of childhood/adolescence relative to the presence of IBD, was used. This data collection method permitted participants sufficient latitude in determining and describing their experiences and the perceived impact of IBD on their daily lives. Ethics approval was obtained prior to study commencement.

### Semi-structured interview guide

Impact of Illness on Self

▪ What is life like for you?

▪ What is life like for you having IBD?

▪ What is the effect of IBD on your life?

▪ When you look at yourself, what do you see?

Functional Impact

▪ How have the things you do changed with IBD?

▪ What activities have changed since your diagnosis?

▪ What do you think of this?

Events/Activities of Importance

▪ What parts of your life are most important to you?

▪ What things do you most enjoy about your life?

Challenges/Obstacles

▪ What parts of your life are most difficult?

▪ What makes your life more difficult than it could be?

Relationships/Family/Peers/School

▪ How has IBD affected you in your family/with friends/in school?

▪ How are things in your family/with friends/in school?

▪ What makes things in your family/with friends/in school easier/more difficult than they could be?

Interviews were subjected to content analysis, concept saturation and theme generation, using 'long interview' qualitative techniques [18]. Key concepts, categories and emerging themes were deduced from participants' comments. According to McCracken's well-established comprehensive analysis approach data analysis comprised the following sequential procedures.

1. Line-by-line coding seeking notable observations in the text that address the research questions;

2. Developing each observation in and of itself from evidence within a single transcript through data categorization;

3. Examining the interconnection of observations in subsequent transcripts through inter-transcript categorization. By this point, themes and an organizational schema was emerging;

4. Emergent themes were scrutinized in collective form for "patterns of intertheme consistency and contradiction" (p. 42). Redundancy was eliminated and themes were organized theoretically. By this time, the focus was no longer on particulars of individual perspectives, but rather on principles and "properties of thought and action within the... group under study" [18].

Rigor was sought in seeking to remain true to the data via the following processes outlined by Lincoln and Guba [19]:

1. Multiple reviewers analyzed the transcripts in determining emerging notions and categories. Discussion and consensus among reviewers followed in formulating emerging themes.

2. Negative case analysis was conducted in revising emerging hypotheses prior to drawing conclusions from the data. As hypotheses were formulated, they were systematically checked against the participants' narratives.

3. Findings were presented to a multidisciplinary group of health care professionals working with this population. Emerging themes were thereby assessed for credibility according to clinical understanding and interpretation.

4. Thick description was implemented in which substantial transcript quotes were used in verifying findings on the basis of participants' verbatim narratives.

## Results

Children described a range of experiences and perceptions about their life with IBD. Common experiences, described by participants, comprised: 1. concerns relating to IBD symptoms and treatments; 2. vulnerability and lack of control; 3. perceiving the self negatively and as different than peers; 4. benefits of social support; and 5. personal resources in coping. While the experiences described were not universal for all participants it was typically found that children with more severe symptoms were more likely to express greater psychosocial difficulty. Each of these commonly-reported experiences is outlined below, along with verbatim quotes (in italics) that demonstrate their application within the interviews.

### 1. Concerns relating to IBD symptoms and treatments

When asked how IBD affected their lives, participants described a range of perspectives. When quiescent, IBD had less effect on day-to-day activities relative to times of symptom exacerbation. Discomfort and concern associated with symptoms and treatments of IBD 'flare-ups' were frequently described. Flare-ups (times of symptom exacerbation) brought disruption in family life, social activities and interrupted school attendance largely due to the child/adolescent feeling unwell, requiring clinic visits and, in some cases, hospitalization. Disruption in daily activities during flare ups, resulted from episodic pain. Bothersome disruptions included pain resulting in mobility limitation and nausea. For instance, a participant stated, *"I can't move or talk when my stomach hurts"*. Another reported, *"The pain is like a knife being stuck in my stomach"*, and *"When my stomach hurts, I throw up"*. Concerns often were heightened in adolescent years and at times when disease severity was greater.

Children often described struggling with food restrictions associated with bowel rest: *"I couldn't stand being away from food. No one realizes how much they like food until they cannot eat"; "I miss getting full when I eat"; "It's hard not to eat"*. Not eating was viewed by some as a restriction from normal social behavior: *"I have to think about every little thing I eat, while others do not"*. Several felt excluded from family rituals such as meals or other *"fun times" *associated with eating. One child stated that *"tube feeding" *left her too embarrassed to be in public, hence she remained in her home.

Exhaustion and malaise were also commonly described: *"You have no energy"; "I'm sick and tired"; "The treatment is frustrating even though it is helping"*. Medications were frequently described as undesirable: *"All the medications I have to take bug me"*. Referring to his medications, one child stated, *"Prednisone makes our life hell. All of us young children would be grateful... (if you could find new medications) because kids can be cruel nowadays. They don't understand what we're going through." *One adolescent stated that medications and the effects of treatments were worse than IBD symptoms. Several felt that the symptoms and treatments required them to sacrifice other opportunities and priorities in their lives: *"I feel like I am missing out"; "It's hard to suspend my life"; "It has stopped my life"*. Along with negative impacts of symptoms and treatments, participants experienced vulnerability and reduced control over many elements of their lives. Given the pervasive and disruptive effects of IBD symptoms and treatment, it is not surprising that disease severity has been found to negatively impact health-related quality of life [20].

### 2. Vulnerability and lack of control

Participants commonly described a personal sense of vulnerability: *"My body is weak internally"; "I am more susceptible to infections"*. The disease and its chronicity was described, in varying degrees, to elicit uncertainties about what these children/adolescents' lives would hold both on a daily basis (e.g., *"I'm often feeling ill, then its hard to eat, get up and concentrate"; "I never know when I'm going to get fat again. It's awful"; You never know what is going to happen"*), and in the future, as illustrated in the following comments.

"I really wanted to be a doctor, but high stress rules that out for me (because of IBD)."

"I fear I won't be able to have kids because I am so skinny, I fear they will never find a cure."

- - - - -

"I don't know about living a normal life. It upsets me... I worry about having to take time off at work."

- - - - -

"I wish I could do things, but I don't do anything. When you are not healthy, you can't do anything."

Participants perceived limited control over their lives due to IBD, particularly intense during disease exacerbation. However, they generally described decreased control in two areas. First, they lived with the possibility that their disease could flare-up at any time; *"I never know when I'm going to have the pain"; "I was so afraid I would throw up, I couldn't leave the house"*. One child described her family as continually, *"on the edge" *– which she described as nervous and tense due to not knowing when a flare-up might occur. Several participants described similar tensions within their families; sometimes intensified by what was viewed as *"over-reaction" *by parents to IBD symptoms: *"My mom goes overboard when I get a flare-up"*.

Second, limited control over participants' lives was experienced due to repeated intrusions upon personal privacy as a result of IBD. Participants' bodies were subject to ongoing surveillance, examination and intrusion by others. In the words of one adolescent, *"IBD makes me feel frightened. I know what they are going to do, but do I really want them to do this to my body?... I feel like I have no control over my own body whatsoever"*.

Of particular concern to several adolescents were issues of privacy particularly in toileting and hygiene. One adolescent stated, *"My mom comes by when I'm on the toilet to see what is wrong. I don't want to ask for help. It's uncomfortable to have my mom watch me on the toilet"*. In this case, intrusion in personal matters violated control and privacy, leaving the adolescent *"uncomfortable" *or *"embarrassed"*.

### 3. Perceiving the self negatively as different than peers

While most participants engaged in routine events at home, school, and with peers, many viewed their life as "different" from the norm. For instance, one adolescent commented on her feelings of being different, stating, *"I'd like to be normal. Normal is not having pain"*. As she experienced pain, she could not view herself as 'normal'. Participants stated that they were reminded of their differences: (i) when they compared themselves to peers (e.g., *"I'm thinner, pale, my hair is thinner. I don't like how I look now. I look sickly"; "Everyone else is growing, but I'm shrinking"; "I want to be socially acceptable, but the medications make me break out and lose weight"*); and (ii) when they had difficulty engaging in activities deemed typical among peers: (e.g., *"When I get sick, I just can't go out"; "One part of you wants to go out, but the other part says it is a waste of time because you will not feel good"; "They've name-called me to death saying 'fat girl'"; "I can never go out"*). Differences were particularly noted among older children in adolescence. Many adolescents, particularly males, described frustration over not being as tall, fast or strong as peers: *"Other kids always yell at me when I miss the ball in sports"*. Several missed activities and outings with peers because of a lack of energy which, for some, left them with fewer friends. In contrast, female adolescents more often expressed concerns over weight gain or cushingoid features associated with treatments.

A common area of struggle described by participants consisted of peer relationships which often included peer judgments, criticisms and sometimes aggressiveness directed toward the child/adolescent with IBD: *"I've been called 'cancer boy' because of thin hair"; "Kids make fun of my problems"; "A group of people push me around"*. With the onset of IBD, many participants described diminishing the amount of time spent with friends. Several commented that their involvement with friends lessened because of: (i) the physical symptoms of IBD (e.g., fatigue, feeling sick); (ii) embarrassment over their appearance, the possibility of going to the bathroom in public places, or passing gas (e.g., *"Being embarrassed (about going to the bathroom in public washrooms) is the worst thing"*; and/or (iii) feeling left out or ostracized by peers: *"I feel isolated and ridiculed"*.

Many described withdrawing from others to avoid negative peer comments and judgments, and in some cases, to squelch personal feelings of being different. For instance, one boy who was receiving nasogastric feeds stated, *"I don't like inviting people over. I'm embarrassed and hide in the closet"*. The tube in his nose left him feeling different and embarrassed. Another adolescent commented, *"I never wanted anyone to come close to me" *because in her words, *"my friends thought I was dying"*. Another stated, *"I don't want to hear from friends because I'm not part of it. They're getting on with their lives, like boyfriends and parties, and I'm going through a horrible time"*. Others expressed concerns about the perceptions and reactions of peers: *"I'm afraid to tell my friends about the disease. I'm afraid they will laugh", "I don't want friends to make fun of me"; "People are scared off when they hear the word, disease"*.

While feeling "different" from peers, participants often wished they could be similar, or as one child stated, *"I just want to be treated like anyone else"*. In contrasting their lives to healthy peers, participants sometimes felt less adequate and/or less privileged: *"I bet other kids don't have any problems"; "I am so different because I have a strange disease"; "If I want to play football, I'm slower"; "IBD makes me different from others"; "I want to be thinner like other kids"*. For several, these "differences", associated with IBD, left them feeling angry: *"I get so embarrassed and angry, I wish I could hit others"; "I'm angry because I'm sick and I can't do what I want to"; "Prednisone made me fat. I'm angry more often because others make fun of it"*. Others expressed emotions of sadness or depression: *"I cry myself to sleep"; "I'm always in a bad mood"; "I get very depressed", "It makes me feel bad and puts me in a slump"; "I just burst out and start crying"*.

Several participants based their view of self on their interpretation of others' perceptions i.e., they felt that others viewed them as a "different" child/adolescent because of IBD. Based on this assumption, they sought to modify parts of themselves perceived as defective or undesirable: *"Others wouldn't make fun of my pudginess if I would exercise"*. In this case, the adolescent viewed herself according to others' perceptions. She identified herself as *"irregular" *based on peer criticisms, and subsequently shaped goals for herself (e.g., increased physical exercise and weight loss) based on these premises.

### 4. Benefits of social support

Participants commonly reported that support from family and friends was a positive influence in coping with IBD. One adolescent stated, *"it helps a lot to talk about (IBD) with someone who has it"*. Another thought that support and encouragement from family members enabled him to experience greater hope. Based on others' *"listening and guiding"*, he had come to feel that, *"I'm glad I have (IBD) because when things happen, they happen for a reason"*. He attributed the support of his parents to his ability to re-frame IBD as something that, *"has made me a special person"*.

Another adolescent felt that the support of her parents had strengthened their relationship, and credited IBD as an opportunity for her parents to demonstrate their love and support. Similar to several participants, she felt that IBD had made her family increasingly cohesive and supportive: *"It has brought my family closer together"*.

When IBD was discussed within participants' families in supportive, age-appropriate and sensitive ways, participants felt more able to openly discuss questions and worries with parents. Accordingly, parents were often characterized as important in how participants viewed and experienced IBD. As an example, one adolescent commented on her parents' openness with her, and its mediating effect on her coping: *"Because my parents and I talk things through about my IBD, I can deal with it"*. As this participant reminisced, she appreciated the openness, *"safety"*, and freedom to discuss important issues such as IBD and its effects with her parents. Other participants, who similarly felt their parents were supportive and honest with them, valued this interaction and commonly felt that they could consequently deal more effectively with IBD. The perceived benefit of this supportive family environment seems important given the periodically-described embarrassing and/or personal nature of issues associated with IBD symptoms and treatments. Thus, relative to some other chronic conditions, the private nature of bowel symptoms may have rendered these young persons more isolated and less comfortable sharing sensitive issues of concern. Accordingly, families that permitted and invited open expression of sensitive issues seemed particularly beneficial to children/adolescents in promoting adaptation.

Conversely, when IBD and its impact were either *not openly discussed *or *not discussed in a sensitive manner*, participants appeared to have greater difficulty coping with IBD. One adolescent described numerous family relationship difficulties and a lack of support from family members. When describing her perceptions about the family's IBD discussions, she stated, *"My family tells me I'm too skinny, and we fight"*. How family members communicated their concerns about this adolescent's situation did little to support her, or foster a positive parent/child relationship. Another child, who characterized his parents as, *"consum(ing) their lives worrying about me"*, and *"uptight" *simultaneously viewed his own life as hopeless: *"everything is pointless"*. The concern and care of his parents, presented in what was viewed to be an overbearing manner, was deemed not helpful to his ability to cope with IBD.

Another adolescent stated that her parents, *"ask questions about what (she) eats all the time"*. This constant surveillance was not viewed as supportive, but rather, excessive. Further, she stated that, *"they call me a marshmallow, and pull on my cheeks when I'm on prednisone. This bugs me"*. Excessive surveillance and a lack of age-appropriate parent/child interaction, as illustrated here, were deemed by participants as not helpful.

Other children/adolescents stated that they could not openly discuss IBD in their families to the extent desired sometimes because they were worried about their parents and or families: *"I am constantly thinking about it (IBD)... but it makes me feel guilty because my family worries about me"; "I would have liked someone else to have been there for me"; "I don't want to make a lot of noise... (because) I'm a burden on my family"; "My parents pretended it was okay... I was hoping it was not anything bad... (so) I didn't want to bring it to my parents' attention"*.

It appears from these data that social support, particularly from family members, is an important resource for children and adolescents in coping with IBD. Effective parental support, based on participants' descriptions, consists of parental attunement to IBD and its symptoms/treatments, as well as to the developmental and individual needs and nuances of the child and adolescent. This includes information and emotional support presented in an environment of honest, age-appropriate and sensitive communication.

### 5. Personal resources in coping

Several children and adolescents described numerous means of coping with IBD; however, this often emerged over time and with maturation. Some recognized new insights and benefits from IBD: *"I now know that if something goes wrong, I have to deal with it"; "I'm more sympathetic to others"*. Some viewed the illness as a means of developing personal integrity: *"I'm a gentler person now"*, and maturity: *"The disease matures you. You act older than you are"*. Several children described hope as a means of adjusting: *"I hope they find a cure soon. Then I won't need to take as many pills"*, and others described a positive or accepting attitude: *"I just take things as they come and make the best of them. Therefore (problems) don't emotionally upset me"; "If people don't accept me, then they don't accept me"*.

Some treated adaptation as a challenge to conquer: *"This disease can rule you, or you can rule it"; "IBD won't get in my way to be an engineer"*. Contrasted to others who were resigned to the struggles of IBD (e.g., *"It stopped my life"; "There is no end in sight"*), this strategy proved effective as a means of pursuing hope and reviving energy to deal with the challenges of IBD. These findings are congruent with previous research which suggests that adolescents who maintain a positive outlook and employ predictive cognitive control as a means of coping with IBD are found to have a better health-related quality of life [21]. Others normalized their experiences with IBD: *"I've gotten used to (IBD) so that it is a part of my life"*. Several used comparison to lessen negative impact of IBD. For instance, an adolescent minimized her struggles by comparing herself with others: *"In coming to the hospital, you see a lot worse off than I am"*. This process of re-framing IBD served to render the challenges more manageable.

Several participants focused on other areas of their lives in which they excelled: *"Some kids look up to me because I am actually pretty smart"*. Others accommodated IBD by finding enjoyable activities that fit within the restrictions imposed by IBD. Some adolescents, for instance, developed interests in handicrafts which they found enjoyable and achievable whether well or unwell, at hospital or home. Using these various strategies, participants persevered and interacted within their families and social worlds. Most felt that they were effectively managing their daily lives and the challenges they faced, and stated that overall, they enjoyed their lives.

## Discussion

Participants conveyed many issues of concern as well as strategies in adapting and responding to IBD. Many struggled with restrictions and challenges, but several also derived vicarious benefits associated with IBD. Some felt that they had matured more rapidly and developed new abilities and interests because of IBD.

Difficulties encountered by the participants in this study included feeling unwell, personal vulnerability, lack of control, diminished self perception and less social interaction. It is difficult to determine, from these data, the extent to which typical child and adolescent developmental struggles were a part of these participants' difficulties. For instance, the extent to which a sample of healthy children and adolescents would similarly dislike elements of their own appearance such as height and weight is unknown. Also, differences in age and gender appear to have a bearing on the emphasized concerns of participants. Adolescents more commonly disliked their appearance than did latency-age children. Latency-age children less commonly and intensely conveyed adjustment concerns. Boys tended to be concerned about reduced strength and short stature more than girls. Girls were more often concerned about weight gain. These areas of concern appear to correspond with gender-based patterns of socialization such that young males may be more concerned about height and athletic competence whereas young females may tend to be more concerned about weight.

Notwithstanding these apparent commonalities with healthy children and adolescents, these findings also identify exaggerated and additional issues for children and adolescents with IBD. As such, IBD may accentuate and complicate typical developmental issues for children and adolescents perhaps more than fundamentally changing the issues of predominant concern.

Perhaps more poignantly, the symptoms and treatments of IBD detrimentally and, in some cases, drastically affect key and sensitive areas of adolescent development. Personal appearance and peer relationships, for instance, appear to be areas that are directly affected by IBD. As such, these are key areas of vulnerability for adolescents that are coincidentally affected by IBD. Thus, IBD negatively targets sensitive issues and developmental areas in which adolescents, as a group, may be most vulnerable and sensitive.

Following this hypothesis, it would make sense that IBD-associated worries (e.g., personal appearance, height, weight) are more pronounced among adolescents than younger children, as was demonstrated in this study. Further, these findings support the chronic illness literature which suggests that illness-specific and contextual factors are linked with overall functioning and quality of life [9,20,22,23]. Quittner and colleagues [23] link parent functioning to ill children's disease-related and psychosocial functioning; however, the findings of the current study further posit that illness-specific issues have an impact on children's functioning. These data extend this argument by suggesting that beyond the need to account for illness-related factors in assessment and potential intervention, developmental level and gender-based factors of ill children also may need to be considered.

In easing some of the concerns and stresses of children and adolescents with IBD and promoting enhanced psychosocial adaptation, family communication and social support were noted to be beneficial. These findings are consistent with a recent study that found that children and adolescents who attended an IBD summer camp experienced improved social functioning, improved total quality of life and better acceptance of IBD symptoms. In this case, attending camp with others who have IBD, boosted social support and normalized the illness experience [24]. Open family communication about IBD and its symptoms and treatments seemed to increase participants' perceptions of personal coping, perhaps by lessening social isolation, increasing feelings of control, permitting IBD-related family dialogue, and allaying unfounded fears and uncertainties about IBD. These findings appear to concur with Maunder and Esplen's [8] conclusion in indicating that the availability and flexibility of support may be beneficial in offsetting adjustment difficulties associated with IBD. Accordingly, family communication and support to the child seem helpful to the extent that they are provided in an age-appropriate manner and within an atmosphere of trust and respect. Questions emerging from these findings include: 'what factors foster or limit effective family communication of IBD-related issues?'; 'what distinguishes families that are able to provide such support from those that are not able to do so?'; and 'how can effective family communication and support be facilitated or encouraged by health care providers?'.

In terms of clinical practice, families in which a child or adolescent has IBD may need to be assisted in re-negotiating patterns of communication and support. Individual family members and, in particular, children and adolescents with IBD may benefit from professional interventions in negotiating, soliciting and participating in family communication and support given heightened risks of family strain and stigma in the pediatric IBD population. Family meetings, psycho-educational materials and/or parent/family workshops addressing effective communication, supportive family interaction, child/adolescent development and/or IBD-specific issues may be beneficial. Access to web-based information and support may augment clinical interventions and provide heightened convenience and anonymity for families in securing needed information and potential peer support.

## Conclusion

The complex psychosocial issues confronting these children and adolescents, such as those emerging from this study (symptoms and treatments, vulnerability, personal control, feelings of being different, coping strategies), need to be recognized in clinical assessments and outcome measures. To the extent that social work and health care practice can assess and effectively respond, the well-being of children and adolescents with IBD can be promoted.

## Competing interests

The author(s) declare that they have no competing interests.

## Authors' contributions

DN predominantly drafted the manuscript, with opportunity for revision by other team members. AMG was the principal investigator on this study. ARO provided leadership in data analysis. CS, MM, and JA assisted in data collection.
